# Efficacy and tolerability of Entecavir for hepatitis B virus infection after hematopoietic stem cell transplantation

**DOI:** 10.1186/2193-1801-3-450

**Published:** 2014-08-20

**Authors:** Jun Aoki, Kiminori Kimura, Kazuhiko Kakihana, Kazuteru Ohashi, Hisashi Sakamaki

**Affiliations:** Hematology Division, Tokyo Metropolitan Cancer and Infectious Diseases Center Komagome Hospital, Tokyo, Japan; Hepatology Division, Tokyo Metropolitan Cancer and Infectious Diseases Center Komagome Hospital, 3-18-22, Honkomagome, Bunkyo-ku, Tokyo, 113-8677 Japan

**Keywords:** Hepatitis B virus, Hematopoietic stem cell transplantation, Entecavir

## Abstract

**Introduction:**

Hepatitis B virus (HBV) flare is a serious problem following hematopoietic stem cell transplantation (HSCT), and the mortality rate is high if severe hepatitis occurs.

**Case description:**

Although Entecavir (ETV) is a standard antiviral drug for HBV infection, the efficacy and safety of ETV therapy in HSCT are still unclear.

**Discussion and Evaluation:**

To examine the efficacy and tolerability of ETV treatment in HSCT, we retrospectively identified 5 patients who received ETV for treatment of HBsAg carrier among patients undergoing HSCT in our institute. We reviewed their clinical information such as clinical course of serum HBV DNA levels, administration period and dose of ETV, and adverse events. There were no episodes of HBV flare or reactivation after HSCT in all patients during the observation period, as a 10-fold rise in HBV DNA levels or positive conversion of HBsAg were not observed.

**Conclusion:**

ETV monotherapy is effective and safe for HBsAg carrier patients following HSCT.

## Introduction

Hepatitis B virus (HBV) flare and reactivation after hematopoietic stem cell transplantation (HSCT) is a life-threatening complication in patients with HBV infection (Liang et al. 1999). HBV related hepatitis is generally observed in hepatitis B surface antigen (HBsAg)-positive and/or HBV DNA-positive patients (Hui et al. 2005). Recently, however, HBV reactivation was reported in patients with even though resolved HBV infection that was indicated negative HBsAg and positive anti-hepatitis B core antibody (HBcAb) and/or HBsAb) at a lower rate (Knoll et al. 2004). It is well established that the frequency of HBV reactivation is higher in HSCT patients (Hammond et al. 2009). The underlying mechanism of HBV flare and reactivation following HSCT is likely to be related to impaired cellular immunity caused by prior chemotherapy and conditioning regimens. Furthermore, administration of immunosuppressive agents, including calcineurin inhibitors and steroids for graft-versus-host disease (GVHD), may exacerbate HBV replication (Xunrong et al. 2001).

HBV-infected patients at high risk of HBV flare and reactivation are recommended to receive preemptive antiviral therapy following HSCT. Lamivudine (LAM), an analogue of cytidine, is available for antiviral therapy in HSCT, (Tomblyn et al. 2009) and inhibits HBV reverse transcriptase, resulting in suppression of HBV replication. Several studies have reported the efficacy of LAM treatments in HSCT patients (Hsiao et al. 2006; Giaccone et al. 2010).

However, LAM treatment has the potential to induce development of drug-resistant mutations owing to the low genetic barrier. Since a small number of mutations are required for LAM resistance, the incidence of LAM-resistant HBV has recently been increasing, and LAM resistance is associated with a rebound in viral load and hepatitis. In fact, LAM treatments showed high resistance and recurrence rates in patients with chronic hepatitis B infection (CHB) undergoing liver transplantation (Perrillo et al. 2001; Mutimer et al. 2000; Lo et al. 2001). Similarly, the appearance of drug-resistant mutants and HBV DNA breakthrough within LAM treatments has been reported in HSCT patients (Hsiao et al. 2006). Thus, more effective agents with lower resistance rates are required in both liver transplantation and HSCT.

Entecavir (ETV), a cyclopentyl guanosine nucleoside analogue, has been approved for treatment of patients with CHB. ETV inhibits reverse transcriptase, DNA replication and transcription. Compared with LAM, ETV has greater antiviral potency and a higher genetic barrier to resistance (Lai et al. 2002). Several studies have shown the superiority of ETV treatments in liver transplantation (Xi et al. 2009; Fung et al. 2011), however, ETV treatment for patients undergoing HSCT has not been reported, and its role in HSCT remains unclear. Here, we describe HBsAg carrier patients administered ETV for treatment following HSCT, and consider its efficacy and tolerability.

## Patients and methods

### Patients

This study was approved by the local medical ethics committee of Tokyo Metropolitan Komagome Hospital. We retrospectively identified HBsAg carrier patients (serum HBsAg-positive and HBV-DNA positive) who received ETV for prophylaxis of HBV flare among patients undergoing HSCT between September 2006 and August 2011. We Laboratory data and clinical information were obtained from our institution’s electronic medical records.

### Hematopoietic stem cell transplantation methods

In our institution, myeloablative conditioning regimens were administered to allogeneic recipients aged <60 years, while elderly patients received fludarabine-based reduced-intensity regimens. GVHD prophylaxis usually comprised short-course methotrexate and cyclosporine A (CsA) or tacrolimus (FK506). For acute GVHD treatment, methylprednisolone (mPSL) 2 mg/kg i.v. in divided dose daily was administered. Antibacterial prophylaxis was provided by tosufloxacin. Steroid-resistant GVHD was treated with a steroid pulse (mPSL 1000 mg i.v. in divided dose daily for 3 days) or mycophenolate mofetil (MMF) 1000 mg twice daily p.o.. Antifungal prophylaxis consisted of fluconazole or itraconazole. Acyclovir or valacyclovir was administered for herpes simplex virus prophylaxis and ganciclovir or foscarnet was administered against cytomegalovirus reactivation.

### Entecavir therapy

ETV 0.5 mg once daily p.o. was administered to patients with HBsAg-positive as primary treatments. The ETV dose was adjusted according to the kidney function. None of the patients received LAM or hepatitis B immunoglobulin at the time of transplantation or during the post-transplant period.

### Hepatitis B virus assay

The levels of HBsAg, anti-HBc and anti-HBs were determined using commercially available chemiluminescence enzyme immunoassay kits (LUMIPULSE Presto HBsAg, LUMIPULSE Presto HBsAb-N, LUMIPULSE Presto HBcAb-III; Fujirebio, Tokyo, Japan). The cut-off index (COI) of the assay of HBsAg, anti-HBs and anti-HBc was 1.0, 10 mIU/mL and 1.0, respectively. The serum HBV DNA concentrations were quantified using the COBAS AmpliPrep/COBAS TaqMan HBV Test (Roche Diagnostics, Basel, Switzerland). The four major HBV genotypes (A–D) were determined by enzyme-linked immunosorbent assay with monoclonal antibodies directed against distinct epitopes on the preS2-region products using commercial kits (HBV GENOTYPE EIA; Institute of Immunology Co. Ltd., Tokyo, Japan) (Orito et al. 2001). HBV DNA sequences bearing the core promoter and precore or core regions were amplified by PCR with heminested primers. The PCR products were directly sequenced by the dideoxy chain termination method using a Big Dye Terminator (Applied Biosystems, Foster city, CA) and an ABI PRISM 3100-avant analyzer (Aritomi et al. 1998).

## Case description

### Patient profiles

**Figure 1:**
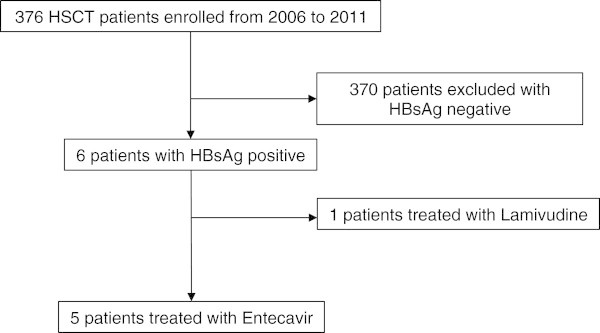
**Flow of study participants.**

Among the 376 patients who received HSCT in our hospital between September 2006 and August 2011, six HBsAg-positive and HBV-DNA positive patients were identified (Figure 1). Of those patients, five patients received ETV for prevention of HBV flare (Table 1). Four patients were inactive chronic hepatitis B (ICHB) (#1, #2, #4 and #5) and other patients were CHB (#3), respectively. Patient #5 has developed HBV reactivation during pre-transplantation therapy at previous treated hospital. Four patients underwent allogeneic HSCT, while one patient underwent autologous HSCT (#1). Within 4 allogeneic recipients, patient #4 received a graft from a matched related donor, while the other three patients received grafts from matched unrelated donors. All of donors were serum HBsAg negative. Myeloablative conditioning regimens were administered to three allogeneic recipients and a fludarabine-based non-myeloablative conditioning regimen was administered to the remaining allogeneic recipient. For GVHD prophylaxis, one patient (#2) received CsA and methotrexate and three patients received FK506 and methotrexate. At the time of transplantation, two patients had undetectable serum HBV DNA levels. Three patients were HBV genotype C and one patient was genotype B. In addition, HBV core and precore promotor mutations were not detected in all patients (Table 2).

**Table 1:** **Characteristics of 5 HSCT recipients treated with Entecavir**

Case	Age (y)	Sex	Disease	Transplantation	GVHD prophylaxis	Steroid administration	cGVHD	Conditioning regimen	Observation period	Sstatus	Cause of death
#1	64	F	MM	Autologous	(−)	No	-	Mel	2 M	Dead	MM
#2	55	F	MDS-RAEB	Allogeneic	FK + MTX	Yes	Yes	BU + CY	9 M	Dead	Aspergillus Pneumonia
#3	24	F	MDS-RCMD	Allogeneic	FK + MTX	Yes	Yes	BU + CY	50 M	Alive	-
#4	58	F	AML	Allogeneic	CsA + MTX	Yes	No	BU + CY	4 M	Dead	Toxoplasmosis
#5	63	F	T-LBL	Allogeneic	FK + MTX	Yes	No	Flu + Mel + TBI(4Gy)	16 M	Alive	-

*Abbreviation: MM* multiple myeloma, *MDS* myelodysplastic syndrome, *RAEB* refractory anemia with excessive blast, *RCMD* refractory cytopenia with multilineage dysplasia, *AML* acute myeloid leukemia, *T-LBL* T lymphoblastic lymphoma, *FK* tacrolimus, *CsA* ciclosporin A, *Mel* melfalan, *BU* busulfan, *CY* cyclophosphamide, *TBI* total body irradiation, *Flu* fludarabine.

**Table 2:** **HBV markers of 5 HSCT recipients treated with Entecavir**

Case	Genotype	HBsAg	HBsAb	HbcAb	BCP/Precore	Initial serum HBV DNA (xLog Copies/ml)	Flare after HSCT
#1	ND	(+)	(−)	(+)	ND	4	No
#2	C	(+)	(−)	(+)	ND	Undetectable	No
#3	B	(+)	(−)	(+)	Wild type	>7.6	No
#4	C	(+)	(−)	(+)	Wild type	Undetectable	No
#5	C	(+)	(−)	(+)	Wild type	7.4	No

*Abbreviation: BPC* basal core promoter, *ND* not determined.

### ETV administration can prevent HBV flare and reactivation following HSCT

The median observation period was 12.5 months (range, 2–50 months). ETV 0.5 mg once daily p.o. was administered as primary treatments for HBV infection. The ETV dose was increased after HSCT with patient #4 due to elevation of HBV viral load. Nevertheless all allogeneic-HSCT recipients suffered from mucous membrane disorder, no patient discontinue ETV oral administration. #4 and #5 patients developed renal disorder due to toxoplasmosis and adverse event of FK506 respectively. The ETV doses were reduced according to the kidney function in these patients. ETV was well tolerated in all cases and no patients discontinued ETV during the follow-up period for severe adverse events. There was no adverse drug interaction between ETV and other drugs. Median neutrophil engraftment time was 20 days (range 16–32 days) in allogeneic HSCT patients (#2-5). No cytopenia due to ETV treatment was observed in all patients. At the last follow-up, three patients (#1, #2 and #4) had died and the causes of mortality were aspergillus pneumonia, aggravation of underlying disease and toxoplasmosis, respectively.

In respect to immunosuppressive agent, two patients (#4 and #5) received mPSL 2 mg/kg i.v. in divided dose daily for acute GVHD in a short period. Patients #2 and #3 suffered chronic GVHD and continued to receive systemic steroid (mPSL 0.5 mg/kg and PSL 0.5 mg/kg i.v. in divided dose daily, respectively) and FK506 for the observation period. Patient #2 received a steroid pulse (mPSL 1000 mg i.v. in divided dose daily for 3 days) against exacerbation of chronic GVHD. Nevertheless, steroid treatment continued during GVHD in these patients and HBV DNA was not detected, indicating that ETV effectively protected against HBV flare and reactivation. In patients #3 and #4, MMF was also added for steroid-refractory GVHD.

**Figure 2:**
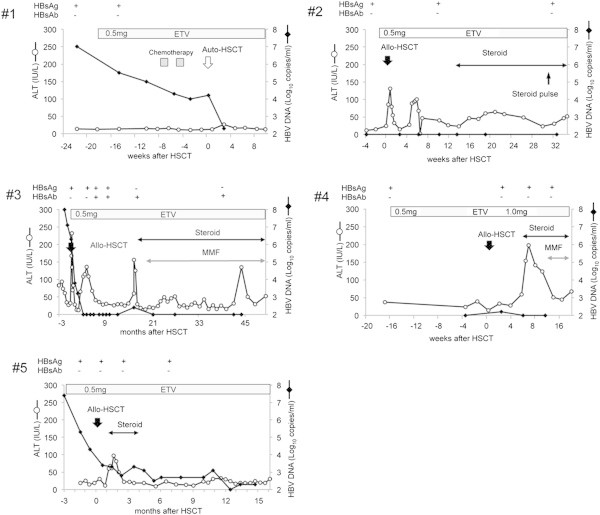
**Clinical courses of HSCT patients treated with ETV.** The X-axis shows the time course, and the Y-axes show the ALT levels and HBV DNA copy numbers, respectively. Transitions of HBsAg and HBsAb are shown at the top. No HBV DNA elevation was observed except for patient #3. HBV DNA of patient #3 became detectable level transiently. ALT elevation after HSCT was observed in all allogeneic HSCT recipients (#2-5). Causes of ALT elevation were considered GVHD or VOD.

There were no episodes of HBV flare or reactivation after HSCT in all patients during the observation period, as a 10-fold rise in HBV DNA levels or positive conversion of HBsAg were not observed. As shown in Figure 2, although patient #3 showed a significantly high HBV DNA level during the HSCT period, HBV flare was not observed. Serum HBsAg was not detected in patient #3 at 18 months after the start of ETV. Subsequently, patient #3 showed reduction of HBV DNA to an undetectable level at the last follow-up.

## Discussion and evaluation

A phase III trial reported that ETV has superior virological, histological and biochemical efficacy as an antiviral drug for HBV infection compared with LAM (Chang et al. 2006). Furthermore, ETV showed a low resistance rate compared with LAM (Tenney et al. 2009). Recent case reports have described successful treatment of HBV reactivation after HSCT with ETV (Christopeit et al. 2010; Milazzo et al. 2011). Therefore, ETV is a strong candidate as a substitute for LAM for HBV prophylaxis in HSCT.

The present study has shown the safety and efficacy of ETV treatment for protection against HBV flare after HSCT. Similar to previous reports for liver transplantation, (Fung et al. 2011) all the patients continued ETV through to the last follow-up without any intolerable adverse events. Intense immunosuppression during HSCT owing to the conditioning regimen and GVHD prophylaxis is a serious risk for HBV flare and reactivation. However, no HBV flare after HSCT was documented during our observation period with ETV treatment. Although blood count after HSCT is not stable, no cytopenia due to ETV was observed. Furthermore, no drug interaction with ETV was documented after HSCT. Our study has demonstrated that ETV treatment is a promising candidate for HBV flare and reactivation prophylaxis in HSCT, similar to the case for liver transplantation.

Seroclearance of HBsAg and HBV DNA was observed with patient #3, despite a high HBV DNA level during the HSCT period. The timing of the serum HBsAg clearance was consistent with that in a liver transplantation study (Fung et al. 2011). Of the 5 patients tested, HBsAb was detectable with 1 patient (#3). The time of detection of HBsAb was 6 weeks after HSCT. HBsAb with patient #3 was detectable during the follow-up period. This positive conversion of HBsAb might be associated with HBsAg clearance (Fung et al. 2011).

All allogeneic HSCT patients showed serum ALT elevation after HSCT. Almost all cases showed ALT elevation without HBV DNA elevation or positive conversion of HBsAg, and we considered that these liver injuries were caused by GVHD or drug-induced hepatotoxicity. Serum ALT elevation and positive conversion of HBV DNA were observed at the same time with patient #3 at 17 months after the HSCT period. However, chronic GVHD symptoms, such as skin keratinization and oral dryness, were exacerbated around the same time and ALT decreased after systemic steroid and MMF administration. Moreover, the positive reaction for HBV DNA was transient and the presence of HBsAg conversely became negative. Collectively, based on these findings, we suggest that the ALT elevation might have been caused by chronic GVHD.

There are some limitations in this study, since it was retrospective and the number of patients was small. Half of the patients died within 1 year after HSCT and their observation periods were insufficient. In future, a large prospective study of ETV prophylaxis in HSCT is required.

## Conclusions

In conclusion, our study showed that ETV monotherapy may be effective and safe after HSCT for patients with HBV. Our study will contribute to future clinical trials.
